# Usefulness of Digital Game-Based Learning in Nursing and Occupational Therapy Degrees: A Comparative Study at the University of Burgos

**DOI:** 10.3390/ijerph182211757

**Published:** 2021-11-09

**Authors:** María Consuelo Sáiz-Manzanares, Caroline Françoise Martin, Laura Alonso-Martínez, Leandro S. Almeida

**Affiliations:** 1 Departamento de Ciencias de la Salud, Facultad de Ciencias de la Salud, Universidad of Burgos, Research Group DATAHES, Pº Comendadores s/n, 09001 Burgos, Spain; 2 Departament Filología Inglesa, Universidad of Burgos, Pº Comendadores s/n, 09001 Burgos, Spain; caroline.martin@ubu.es; 3 Departamento de Ciencias de la Educación, Facultad de Educación, Universidad de Burgos, C/Villadiego, 1, 09001 Burgos, Spain; lamartinez@ubu.es; 4 Instituto de Educação, Campus de Gualtar, Universidade do Minho, Research Group CIEd, 4710-057 Braga, Portugal; leandro@ie.uminho.pt

**Keywords:** higher education, mixed methods, self-regulated learning, academic achievement, gamification, nursing, occupational therapy

## Abstract

Teaching in higher education in the 21st century is moving towards e-Learning or b-Learning teaching models. This situation has increased due to the SARS CoV-2 health crisis. Therefore, teaching–learning models must be based on the use of active methodologies that facilitate students’ motivation to work in learning management systems (LMS). One of the most current resources is the digital game-based learning (DGBL) use, specifically in health sciences degrees (e.g., nursing). In this study, we worked with 225 third-year students of degrees in nursing (ND) and occupational therapy (OTD). The objectives were (1) to find out if there were significant differences between students who had worked with DGBL techniques vs. those who had not, and (2) to find out if there were significant differences depending on the type of degree (ND vs. OTD) regarding access to the LMS, learning outcomes and students’ satisfaction with teachers’ performance. A mixed-method research approach was applied. In the quantitative study, significant differences were found in the accesses to the LMS in favor of the groups that had worked with DGBL techniques. Significant differences were also found in ND students with respect to learning outcomes in the group that worked with DGBL. Regarding the results of the qualitative study, differences were found in the frequency of interaction and in the preference of DGBL activities depending on the type of degree. Further studies will investigate the possible causes of these differences.

## 1. Introduction

The relationship between the use of digital game-based learning (DGBL) applied in learning management systems (LMS) from the use of intelligent tutoring systems (ITS) will be analyzed below.

### 1.1. State of the Art

The use of advance learning technologies (ALTs) in technology-rich learning environments (TRLEs) is playing an increasingly important role in 21st-century education. This has accelerated in the wake of the SARS-CoV-2 situation [1,2]. Recent studies [3] indicate that learning spaces in which ALTs are used guide the development of active learning strategies and positive emotions in students. Specifically, within ALT, the use of game-based training [4] stands out; specifically, it is being used with great acceptance in the higher education stage in health sciences degrees. One of the reasons is related to the need for students of these disciplines to acquire skills focused on clinical practice. This practice must be implemented from the observation and interpretation of data in an exploration. The ultimate goal is to avoid as many errors as possible in the diagnosis and, therefore, in the treatment. To achieve this objective, in recent years, innovative methodologies have been implemented in the training of future professionals in these disciplines. Among these active methodologies, the use of serious games techniques stands out. This use seems to facilitate the use of metacognitive strategies in students, specifically related to planning and self-evaluation of learning outcomes. In turn, these strategies are related to self-awareness about their own learning, increased motivation and student autonomy in during the process. Thus, at the same time, students develop a greater sense of academic responsibility and self-esteem. There are many types of serious games, such as simulation games, presentation games, role-playing games, testing games, etc. [1].

The latest research currently refers to the effectiveness of DGBL [1]. One of the key elements for success is the quality of the pedagogical and technological design of the games. In order to test this, questionnaires or scales of usability and functionality of the proposals for the students are used. Following the proposal of Wynne and Hadwin [5] in DGBL, the following phases must be differentiated: (1) clear definition of the task; (2) planning and goal setting and (3) make a pedagogical and technological design in a learning context as the one proposed by TPACK (Technological, Pedagogical and Content Knowledge framework) (http://tpack.org/ accessed on 2 November 2021) [6]. This approach can be combined with an online project-based learning (OPBL) structure that starts from a research or inquiry question. In the current situation caused by COVID-19, it is proving to be a very effective technique to combine DGBL activities with OPBL [7,8].

Within this framework, current research emphasizes the need to monitor and evaluate DGBL processes by applying scientific methodologies to assess their effectiveness in learning processes [1]. However, some researchers who carry out studies with high scientific rigor in this field are not publishing the results found due to situations of bias in the research approaches [9]. Hence, we must consider that although research studies should be designed with the greatest possible scientific control, educational contexts are not closed laboratories to the impact of multiple procedural and contextual variables. It is difficult to ensure control of all the variables involved in the process. In order to mitigate this problem, many of the research studies in this field are currently applying mixed research methods, i.e., using both quantitative and qualitative designs in combination [10]. Furthermore, the latest trends in research in this field suggest that feasibility studies or pilot studies should be conducted prior to implementing a DGBL experience. A feasibility study refers to a pilot or descriptive research that aims to “test” a certain educational resource with a view to prove its effectiveness and guide a more stringent research [1,10]. The feasibility of an educational resource can also be found using survey studies or meta-analysis [11]. Both feasibility studies and pilot studies have smaller sample sizes [10,11], which makes it difficult to generalize the results but sheds light on possible difficulties and hypotheses to be tested. However, there are few studies investigating the feasibility of the methodology for the evaluation of the efficacy of DGBL due to the bias problems indicated above. Although these studies have a bias component, they are very important in order to advance in the use of this methodology in LMS since they provide the ecological validity of the instructional intervention.

Regarding students’ perception of the use of these innovation techniques, it has been found that DGBL experiences that include automated feedback on results in LMSs [12] facilitate the development of cognitive strategies, metacognitive strategies and positive feelings towards learning [13]. One possible explanation is that DGBL techniques enhance self-regulated learning (SRL) [14].

In summary, the instructional methodology based on technology involves a pedagogical redesign on what, how and when to teach and evaluate [15]. This fact involves an adaptation of the teaching–learning processes to the new educational framework in the 21st-century society [16] which is developed in a very high percentage in virtual teaching–learning spaces such as LMS [17]. In addition, the structuring of information in these virtual spaces must be made attractive to the learner and include elements that enhance the development of SRL [18]. To achieve this, DGBLs need to include quizzes with process-oriented feedback and a progress analysis system [19] in order to facilitate learner autonomy.

### 1.2. Application of the Gamification Technique to Improve Learning

As already indicated, the use of gamification techniques in learning environments has increased in the past two years, especially in higher education. However, there is no consensus in the scientific community regarding a taxonomy of classification of DGBL. Actually, DGBL would be all activities designed with a serious game structure aimed at facilitating learning [20,21]. For example, van Gaalen et al. [22] pointed out that simulation activities would not be proper gamification activities, but rather actions to solve tasks or problems more related to OPBL techniques. The latter are related to an increase in student learning outcomes [23]. Nevertheless, this fact has not yet been individually verified in the use of DGBL. All these active learning methodologies are oriented towards a different “way of doing” rather than those based on more traditional approaches to transmit knowledge. Altogether, innovative methodologies encourage the active participation of the student in the teaching–learning process with the central objective of developing knowledge and skills. In addition, they enable critical thinking and divergent ideas in the student. All of this in itself is an important achievement for the change in the way of teaching in higher education, specifically in degrees that require the implementation of competencies for the practical resolution of situations [23].

Particularly, research studies [15] have shown that the use of DGBL together with the use of automatic feedback on responses facilitates SRL. This design includes the analysis of the user’s learning process through recording and monitoring techniques of different processes within a loop that increases motivation and autonomous learning [24] from the use of automatic feedback, i.e., from the use of ITS [25]. Therefore, the use of SRL together with the use of gamification activities facilitates the development of process-oriented feedback. This type of feedback allows the student to understand the task to be solved (focusing on the objective of the task) and how to solve it (analysis of the strategies to solve it) [26]. All these resources will enhance the development of metacognitive strategies, including self-awareness, self-assessment and metacognitive correction of possible errors [27].

**Figure 1:**
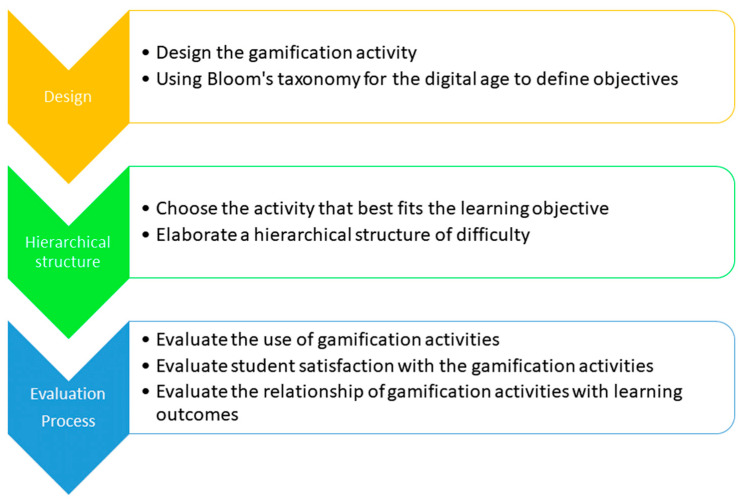
Process for the design and evaluation of gamification activities.

The resources presented can be included in the LMS through the use of hypermedia tools in e-Learning or b-Learning learning spaces. Currently, the teacher with digital competences can design and implement these gamification actions using technology, i.e., HyperText Markup Language Package -HTML-5 (H5P) (https://h5p.org/ accessed on 2 November 2021) [28]. H5P facilitates the creation of interactive content and provides an important variety of activities and resources. Interactive content can be included in WordPress, Moodle (Modular Object-Oriented Dynamic Learning Environment) or Drupal. They can also be integrated into LMS through linear time-invariant (LTI) with Canvas, Brightspace and Blackboard, among others. However, the use of these resources must follow a rigorous design process within TPACK. To do so, first of all, the teacher has to design the activity [29]. An important resource for the definition of the competences to be achieved by the students is the use of Bloom’s taxonomy for the digital age [30]. Then, the teacher must choose the activity that best fits for the acquisition of each competence. Subsequently, he/she has to elaborate a hierarchical structure of difficulty regarding the acquisition of competences and its relationship with the type of H5P activity to be implemented for their achievement. The final objective will be to offer the students different activities with different degrees of difficulty so that the student can progress in the knowledge at their own pace of learning. A summary of this process can be found in Figure 1.

Finally, it is necessary to highlight the difference between gamification, serious play and game-based learning. Gamification could be defined as the use of game elements in non-playful contexts. Serious games would refer to games created ad hoc with the purpose of developing skills or some specific knowledge, and game-based learning refers to technological or traditional games that are intended to facilitate the development of learning [31]. The study will be carried out following the guidelines of the latter approach.

H5P offers different types of resources that help the teacher to implement different gamification activities (see Table A1). The teacher can use the functionality offered by H5P to develop interactive activities that can be applied to practical assumptions [32], flipped classroom experiences [33], simulation games related to problem solving [32,34], escape games [35], etc. All these activities are very useful to apply them to the training of students of health sciences (i.e., nursing, occupational therapy, medicine, psychology, etc.). Specifically, healthcare-based escape room games are being used with great success in nursing degree programs [36]. This type of game facilitates student retention and application of patient care knowledge. Additionally, it has been shown to improve the quality of patient care in real intervention settings [36]. Similarly, it increases student engagement and the development of critical thinking [37]. In addition, it increases students´ motivation and satisfaction with the learning process [38]. Likewise, all these activities allow the teacher to program automatic feedback on the results and progress control. In addition, the use of these resources will allow ITS and autonomy in the control of the process by the student. Recent research [39] has shown that students perceive that performing these activities helps them to improve their retention and understanding of information.

Nonetheless, there is still a long way to go in research in these environments. Vermeir et al. (2020) [40], in a meta-analysis study on the advantages of gamification in improving learning, indicate that progress in this field will depend on conducting research that focuses on:

1. Studying the impact of gamification together with other active methodologies such as OPBL;
2. Describing the design of serious games;
3. Studying the users’ perception of the functionality of serious games in their learning;
4. Theoretical basis for the design of the gamification process; and
5. Analyzing the cognitive and metacognitive consequences of the use of gamification in students. Similarly, in another systematic review study by Malicki et al. (2020) [41], it is evident that there are not enough studies addressing the relationship between the use of gamification and its impact on students learning outcomes.

On the basis of the above, a mixed research method was used in this work. Therefore, two types of studies were proposed: quantitative and qualitative.

#### 1.2.1. Quantitative Study

The following research questions were formulated in this study:

RQ1: Will there be significant differences in platform accesses, average visits per day, learning outcomes, and student satisfaction with the teaching process depending on DGBL technology implementation vs. non-implementation?

RQ2: Are there significant differences in the accesses to the platform, the average number of visits per day, the learning outcomes and the satisfaction of students with the teaching process depending on the type of degree (occupational therapy vs. nursing)?

#### 1.2.2. Qualitative Study

The following research question was formulated in this study:

RQ3: Will different types of feelings (positive, neutral and negative) be found in students regarding the use of DGBL depending on the type of degree they take (occupational therapy vs. nursing)?

## 2. Materials and Methods

### 2.1. Participants

We worked with a sample of 225 students in the third year of health sciences degrees during two academic years. The work in both academic years was carried out during the COVID-19 pandemic situation (academic year 2019–2020 and academic year 2020–2021), with 98 occupational therapy students and 127 nursing students (see Table 1). Convenience sampling was applied for the selection of the sample. In the academic year 2019–2020 DGBL techniques were not used, in the academic year (2020–2021) they were applied.

### 2.2. Instruments

#### 2.2.1. Learning Management Systems Based on Moodle v.3.9: Virtual Learning Platform of the University of Burgos, UBUVirtual

It is a virtual learning platform based on Modular Object Oriented Dynamic Learning Environment (Moodle).

#### 2.2.2. ACRA Metacognitive Strategies Scale by Román-Sánchez and Gallego-Rico

This instrument [42] is highly contrasted in Spanish-speaking populations [43]. ACRA identifies 32 learning strategies at different stages of information processing. In this study, only the Metacognitive Strategies Scale was used. It includes 3 subscales: self-knowledge, self-planning and self-evaluation. The Metacognitive Strategies Scale has a Cronbach’s alpha reliability coefficient of α = 0.90, an inter-rater construct validity of r = 0.88 and a content validity of r = 0.88. Specifically, for this study, values were found for the general scale of α = 0.87, self-knowledge subscale of α = 0.82, self-planning subscale of α = 0.85 and self-evaluation subscale of α = 0.78.

#### 2.2.3. Gamification Activities

The following gamification activities were designed with H5P: crossword, find the words, memory game, speak the words set and true/false question. All these activities included process-oriented feedback on the answer and a progress bar. In addition, they all had three levels of difficulty (beginner, intermediate and advanced).

#### 2.2.4. eOrientation Moodle Plugin

This plugin was developed within a research project funded by the Junta de Castilla y León (Spain) (see Funding). The plugin [44] can be used to configure personalized access to the pedagogical resources used by the teacher in each subject and in each academic year. It also facilitates personalized communication with each student via email. This functionality allows the teacher to give feedback to the student on the results of the monitoring of the learning process. In addition, the logs can be exported in different combinations of the components and in different formats (.csv, .xlsx, HTML table, .json, .ods, .pdf) [45].

#### 2.2.5. UBUMonitor Application

UBUMonitor (Universidad de Burgos, Burgos, Spain) [46] is a free and open access desktop application (https://github.com/yjx0003/UBUMonitor accessed on 2 November 2021). The application connects to the selected Moodle server through web services and the REST API provided by the server. All communication between the Moodle server and the UBUMonitor client is encrypted for security reasons by the HTTPS protocol. As a result of these queries, data are obtained in JSON and CSV format and are processed and transformed in the client into Java objects. UBUMonitor contains five modules: (1) visualization module (allows an analysis of access frequencies in components, events sections or course viewed in Moodle) with options to analyze the logs in different graphs (boxplot, etc.); all visualization options allow export in graphical format and in .csv format for reporting and further analysis with other tools; (2) comparison module, which analyzes students’ records in components, events, sections or course viewed in Moodle, grades and activity completion, and gives information about frequencies from a visual comparison in the ranking and in the analysis of students’ evolution; (3) dropout risk module (gives information by intervals 0–3 days, 3–7 days, 7–14 and more than 14 days) about the access of the students to the course and about the access to the Moodle platform; and (4) clustering module, which finds the clusters from different algorithms (k-means++, fuzzy k-means, DBSCAN, MutiMeans++, etc.) and different distances (Euclidea, Manhattan, etc.) that are processed from two Java libraries. In this study, the visualization and comparison modules were used for the analysis of students’ behaviors in gamification activities on the UBUVirtual platform.

#### 2.2.6. Online Project-Based Learning (OPBL)

All groups of students worked with the OPBL methodology. Students in small groups (3 to 5) members, either in occupational therapy or nursing programs, had to design an intervention from their professional role regarding a practical case that the group had previously chosen. This is the pedagogical design implemented in this research.

#### 2.2.7. Questionnaire of General Satisfaction with the Training Activity 

The questionnaire is an *ad hoc* survey [47] consisting of 19 closed-ended questions measured on a Likert-type scale of 1 to 5 from 1 (do not agree at all) to 5 (strongly agree) and three open-ended questions related to strengths, weaknesses and proposals for improvement. The survey had in this study a Cronbach’s reliability coefficient of α = 0.93 (see Appendix A, Table A2).

#### 2.2.8. Questionnaire of Satisfaction with the Gamification Activities 

This questionnaire is an *ad hoc* survey [48] consisting of one closed-ended question measured on a Likert-type scale of 1 to 5 from 1 (do not agree at all) to 5 (strongly agree) and three open-ended questions about the quality of the gamification activities (see Appendix A, Table A3).

### 2.3. Procedure

Before starting the study, a positive report was obtained from the Bioethics Committee of the University of Burgos (no. IR 30/2019) along with written informed consent of all research participants. We worked at the Faculty of Health Sciences of the University of Burgos with third-year students of the occupational therapy and nursing degree programs over two academic years (first year and second year of the COVID-19 pandemic). The teaching was performed by the same teacher in all groups in order to control the effect of the variable type of teacher. The teaching during the first year of the pandemic (academic year 2019–2020) was carried out in e-Learning mode and DGBL was not applied. Teaching during the second year of the pandemic (academic year 2020–2021) was carried out in b-Learning mode and DGBL was applied. The duration of DGBL in the two degrees (occupational therapy and nursing) was conducted during the last four weeks of the semester and the participation of the students was voluntary. The gamification activities in the two degrees (occupational therapy and nursing) were applied during the last four weeks of the semester. Examples of gamification processes can be found in the Supplementary Materials, Table S1. Participating students could earn up to one point of the final grade in the course depending on their degree of involvement in the evaluation activities. The OBPL methodology was used in all groups. The learning process was also monitored using the eOrientation [44,45] and UBUMonitor [46] tools. The data on the statistical contrasts can be found in Section 2.5, Data Analysis.

### 2.4. Research Designs

A mixed methods research methodology was applied. In the quantitative study, a 2 × 2 factorial design was applied (type of degree (occupational therapy vs. nursing) and application of DGBL vs. non-application) [49]. Analyses were performed with the SPSS v.24 statistical package [50]. In the qualitative study a comparative longitudinal design was used [51]. In this case, the data analysis was performed with the qualitative analysis software ATLAS.ti v.9 [52].

### 2.5. Data Analysis

In the quantitative study, prior to the contrast of the research questions, an analysis of skewness and kurtosis was performed on the scores obtained by all students in the ACRA Metacognitive Strategies Scale [42] applied before the intervention. No extreme indicators of skewness or kurtosis were found, so parametric statistics were applied to contrast RQ1 and RQ2. In addition, a one-factor ANOVA with fixed effects (type of student group) was used to check if there were significant differences before the intervention in the aforementioned scale. Then, to contrast RQ1 and RQ2, a two-factor ANOVA with fixed effects (type of degree, occupational therapy vs. nursing, and application of DGBL vs. non-application) and eta squared effect value was used.

Regarding the qualitative study, a categorization analysis of the responses to the open questions of the Survey of Satisfaction with the Gamification Activities [48] and an occurrence documentary analysis (frequencies and percentages) was carried out. A visualization analysis was also used to compare the behavior of the students of the bachelor’s degrees in occupational therapy and nursing in the performance of the gamification activities. The visualization module of the tool UBUMonitor [46] stacked bar graph was used for this purpose.

## 3. Results

### 3.1. Previous Analysis Quantitative Study

As indicated in Section 2.5, in the quantitative study, before the contrast of the RQs, we first checked the skewness and kurtosis values of the sample with respect to the results on the ACRA Metacognitive Strategies Scale [42]. No extreme values of skewness (see Table 2) (according to Bandalos and Finney [53], extreme values are considered to be those greater than |2.00|) or kurtosis (according to Bandalos and Finney [53], extreme values are considered to be those between |8.00| and |20.00|) were noted. Based on the results, it can be concluded that the sample followed a normal distribution. Therefore, parametric tests were applied to contrast RQ1 and RQ2.

Subsequently, a one-factor fixed-effect ANOVA was performed to test whether before the intervention there were significant intra- and inter-group differences in the results of the Metacognitive Strategies Scale [42]. Significant differences were found between occupational therapy students vs. nursing students in favor of the nursing students on the self-knowledge scale. Likewise, no significant differences were detected with respect to the teaching year variable. Since it was not possible to consider homogeneous groups in all the variables, the contrast of RQ1 and RQ2 was only conducted intra-degree (see Table 3).

### 3.2. Contrasting RQ1 in the Quantitative Study

In order to compare RQ1, intra-group contrasts were carried between the occupational therapy and nursing students.

#### 3.2.1. Results in the Degree in Occupational Therapy

Significant differences were found between the students of occupational therapy in access to the LMS, and the average number of views per day in favor of the group in which DGBL was used, being the mean effect value. No significant differences were found in the learning outcomes (see Table 4).

#### 3.2.2. Results for the Bachelor’s Degree in Nursing

In this group, significant differences were found in access to the LMS, with the average number of views per day in favor of the group in which DGBL was used, with a low effect value. Significant differences were also found in learning outcomes supporting the group in which DGBL was not applied (see Table 5).

### 3.3. Contrasting the RQ2 in the Quantitative Study

With respect to RQ2, no significant differences were found in student satisfaction with teaching in either group with respect to DGBL use vs. non-use.

To check the degree of satisfaction within the groups in which DGBL had been applied, the answers to the closed question and the open questions of the Survey of Satisfaction with the Gamification Activities were analyzed [48]. Regarding the closed-ended question, a mean of 4.28 out of 5 and a standard deviation of 0.22 were obtained in the group of students of the occupational therapy degree program. Similarly, in the group of students of the nursing degree, a mean of 4.29 out of 5 and a standard deviation of 0.55 was obtained. Hence, it can be concluded that the degree of satisfaction of the students with the gamification activities was high in both groups with a very low degree of dispersion, which indicates a high degree of homogeneity in the degree of agreement. Thereafter, the answers to the open-ended questions were analyzed. A categorization analysis of the answers given by the students of the two grades was carried out. Regarding question 2 (“Which of the gamification materials have been most useful for the understanding of the concepts?”), the students of the occupational therapy degree considered that the true/false test-type gamification activities were the most effective for their learning. Likewise, the students of the nursing degree considered the true/false test gamification activities to be the most useful, together with the crossword activities in the same proportion. Regarding question 3 (“What elements would you introduce or increase in the gamification materials?”), 71.42% of the occupational therapy students indicated that they would eliminate the “speak the words set” type of gamification activities, since this tool presented failures in the recognition of voice responses. On the other hand, the nursing students thought that the multiple choice, relationship questions and image games could be increased. Regarding question 4 (“Which of the gamification elements would you eliminate and why?”), both groups indicated that it would be good to eliminate the “find the words” games, as they did not involve any deduction challenge and only involved the discrimination of the word within the grid (see Table 6).

### 3.4. Contrasting RQ3 in the Qualitative Study

To contrast the RQ3, a feeling analysis was performed with the ATLAS.ti v.9 [52] program with respect to the answers to the open questions of the Survey of Satisfaction with the Gamification Activities [48]. ATLAS.ti v.9 [52] allows a text mining analysis of the sentences, classifying them as negative feeling (applied when it detects in the answers a question, negative particles or the introduction of a suggestion), positive feeling (when it detects total agreement or satisfaction) and neutral feeling (when the answers indicate neither agreement nor disagreement). As can be seen in Table 7, more sentences classified as negative feelings than positive feelings were detected in both groups of students.

Some of these sentences are transcribed below so that the type of thinking expressed by students in both groups can be appreciated.

1. Occupational therapy degree. Examples of negative thoughts:

“More activities that require working on definitions of concepts”.

“I wouldn’t eliminate any, but I would make some improvement such as:

-The crossword puzzles are not marked as completed.

-In the word game, at the beginning no problem but as he progresses he doesn’t understand the words well despite saying them correctly many times”.

“I would increase the number of true and false, as I think it can be very interesting.”

“You could include matching activities, for example a word with its definition. Picture games, linking pictures with concepts”.

2. Nursing degree. Examples of negative thoughts:

“I would eliminate the alphabet soup. I don’t see much use for it when it comes to grasping concepts.”

“Perhaps I would eliminate the word search game as I think it is the one that can help us the least with respect to understanding the concepts.”

“The word search game has been the least useful for me although it has been fun. Maybe I wouldn’t remove it but instead of giving the word, I would give the definition of the concept that we should look for and based on that find the word”.

“I would not eliminate any element of gamification as, in my opinion, they facilitate the understanding of the syllabus. I would also modify the word search as we only have to look for the word you give us in it, I would focus it more like the crossword puzzle: you give us a definition of a concept and we look for the word”.

**Figure 2:**
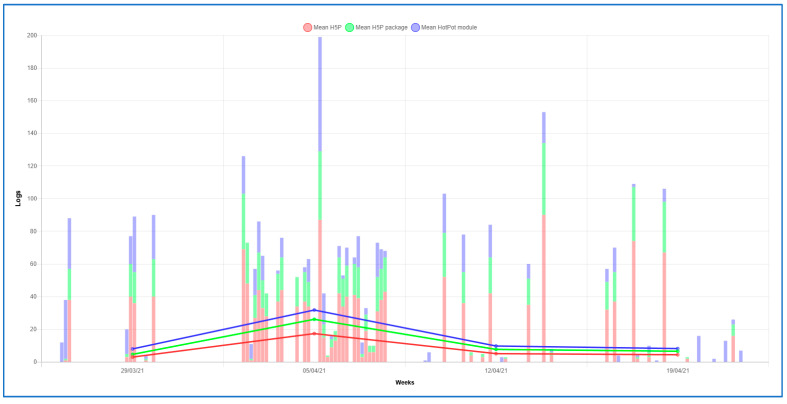
Stacked bar of the interaction records of the occupational therapy students in the DGBL activities.

**Figure 3:**
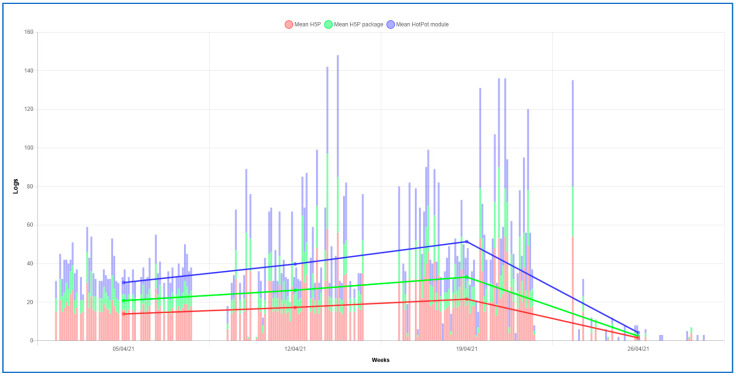
Stacked bar of the interaction records of the students of the degree in nursing in the DGBL activities.

Regarding the pattern of students´ behavior in both grades, it has a similar structure. At the beginning of the activity in DGBL, a lower frequency of these activities was observed, and the highest peak of interaction was reached in the second and third week. Finally, in the last week, a decrease in frequency was observed. However, a higher frequency of activity was observed in the nursing students than in the occupational therapy students (see Figure 2 and Figure 3).

## 4. Discussion

The use of DGBL in LMS is a novel practice especially relevant in the 21st century [15,22,23,24,25,26] and specifically in the current situation with rules of social isolation worldwide due to the COVID-19 health crisis [1,2]. This situation has shown that teaching, specifically in higher education, is being developed in e-Learning or b-Learning spaces, depending on the confinement phase, even for materials to be taught face-to-face. In this context, it is necessary to offer students learning activities that are novel and motivating. The use of DGBL becomes an ideal resource for this purpose [1,3]. In this research, we found that undergraduates can apply specific metacognitive strategies depending on the type of degree. Specifically, students of the bachelor’s degree in nursing obtained higher scores in the use of metacognitive strategies of self-knowledge, both in the group in which DGBL was applied and in the group in which it was not applied. This finding suggests that there are variables such as learning style that may be conditioning a greater or lesser effectiveness of gamification techniques on learning outcomes. As other studies have already indicated [6,8], it is very difficult to isolate the effects of gamification from those of other factors. However, it seems that the use of these techniques increases the interaction of students in the LMS [2]. In this study, an increase in interaction in the LMS was found in the two groups, occupational therapy and nursing students, in which DGBL was applied [2]. Nevertheless, no significant differences were found in students´ satisfaction with teaching depending on the use or non-use of DGBL. One possible explanation is that in all groups, the OPBL methodology was also applied, and this in itself generates indicators of satisfaction [7,8]. In this sense, as previously indicated, the results of this work support those found in other studies that indicate the difficulty of discriminating the unique effects of the use of gamification [6,7,8,23], since both methodological resources, DGBL and OPBL, facilitate the construction of meaningful learning, enhance the use of SRL and increase students´ satisfaction [3,5,12,13,14,18].

Regarding the impact of the use of DGBL on learning outcomes in this study, no significant differences were found in the occupational therapy undergraduate students and significant differences were found in the nursing undergraduate group. These findings support the findings of other research that there is no universal conclusion of the effects of DGBL on learning outcomes in students [41]. In addition, to obtain more information about students’ perception on the effectiveness of DGBL in their learning process, a qualitative analysis was applied in this study [10]. The ultimate aim was to analyze the type of feelings generated by the students’ use of DGBL. The results indicate that the satisfaction was high among students of both degrees in whom DGBL was applied [38,39,40]. At first, very similar percentages were found in the three types of feelings (negative, positive and neutral), with negative feelings having the greatest weight. This fact can be explained, as previously indicated, because the automatic text mining analysis applied by the ATLAS.ti v.9 program when the sentence includes a negative particle or a proposal for improvement classifies it as a negative feeling. However, the interpretation in this study is that these sentences classified as negative feelings offer improvement proposals to increase the effectiveness of the use of DGBL in students [24,25,26]. This conclusion is very important and significant, since the use of gamification activities seems to facilitate the implementation of critical thinking in students [23]. That is, the use of DGBL favors the development of metacognitive strategies of self-evaluation and reflection. This achievement will foreseeably facilitate student achievement of autonomous and deep learning [13,14,26]. Furthermore, this self-reflection leads the student to evaluate which of the DGBL activities used was more functional for their learning and which should be increased or decreased in frequency. This is an important indicator to check in future research, since it may be necessary to design personalized gamification activities depending on the type of student and the area of knowledge. The use of DGBL is a resource with important potential in the research of the teaching–learning process in virtual environments [41].

Nevertheless, the results found in this study must be taken carefully due to the characteristics of the sample, since we worked with third-year students in two degree programs at the same university and the selection was not random. Moreover, this work addressed the analysis of the effects of DGBL, specifically of conceptual fixation games and testing games, but not the effectiveness of simulation games [22,32,34,36,37]. It is proposed to address this study in future research.

## 5. Conclusions

In summary, it can be concluded that the analysis of the effectiveness of the use of DGBL on learning outcomes and satisfaction in higher education students, specifically in health sciences, is a complex process since it requires a precise design and implementation of gamification activities. In addition, these must be aligned with the conceptual and procedural contents that the teacher wants to transmit. On the other hand, the use of gamification activities in LMS environments requires the implementation of technological resources such as H5P technology. This last aspect requires the teacher to be trained in the use of these types of techniques. On the other hand, the effectiveness of different gamification activities is not the same in different groups of students. The teacher will have to test systematically throughout the teaching–learning process which DGBL resources will be the most effective for each group of students. This situation demands a considerable effort that is essential if the teacher wants to achieve a high degree of motivation and the achievement of effective learning in their students. In sum, there is still a long way to go in this novel area of research. To address this challenge successfully, the development of DGBLs needs to involve interdisciplinary teams. Such teams must be composed of experts in the subject matter, DGBL techniques, in technology to implement the DGBL techniques and in data analysis extracted from LMSs. The final objective will be the TPACK-based design of effective gamified learning environments.

It is relevant to note that in this research, many tools were used to monitor the tracking of the undergraduates during the development of the teaching–learning process. This aspect is essential in order to understand how the elements of educational innovation that the lecturer implements are perceived by students. The possibility of monitoring in real time is one of the strengths of this study. The reason is that the effectiveness of the application of gamification techniques may be different for each learner or groups of learners, and this lies in the key to success in its use. Likewise, the research limitations reside in the collection of the undergraduate sample through an intentional and opportunity sampling instead of random probability sampling. However, it must be considered that if these studies want to conduct a detailed monitoring of the effectiveness of gamification techniques, it is advisable to perform the research with samples that are not very large, in which continuous tracking is possible.

To conclude, it is an important challenge to include gamification in the curricula of degrees, such as health sciences, where the use of such resources shown to be effective in clinical practice. Consequently, more research is needed to shed light on the effectiveness of DGBLs on students’ learning outcomes and on the differentiation of their effectiveness with respect to other active methodologies. Thus, we emphasize the need for feasibility or pilot research in order to find answers to the challenges that the use of DGBLs is posing in the current context of higher education.

## 6. Patents

Sáiz-Manzanares, M.C. et al. eOrientation Computer Software for Moodle. Detection of the student at academic risk at University No. 00/2020/588; General Registry of Intellectual Property: Madrid, Spain, 16 January 2020 [44].

## Figures and Tables

**Table 1:** Descriptive statistics in the sample of participants.

Teaching Performance	*N*	Occupational Therapy Degree (*n* = 98)						Nursing Degree (*n* = 127)					
		*n*	Men		*n*	Women		*n*	Men		*n*	Women	
			M_age_	SD_age_		M_age_	SD_age_		Mage	SD_age_		M_age_	SD_age_
Course 2019–2020. Non-application of DGBL techniques	107	8	21.63	1.77	38	22.42	2.25	5	21.40	0.89	56	23.54	6.30
Course 2020–2021. Applied DGBL techniques	118	7	21.57	0.79	45	22.64	4.72	7	25.71	7.39	59	23.44	5.51

Note: M_age_ = mean age; SD_age_ = standard deviation age.

**Table 2:** Descriptive statistics and analysis of skewness and kurtosis in the Metacognitive Strategies Scale [42].

Metacognitive Strategies Scale [42]	M	SD	A	SEA	K	SEK	Score Maximum
Self-knowledge	20.04	2.36	−1.22	0.16	4.01	0.32	28
Self-planning	12.53	1.97	−0.98	0.16	2.84	0.32	16
Self-evaluation	19.28	2.31	−1.01	0.16	4.18	0.32	24

Note: M = mean; SD = standard deviation; A = skewness; SEA = standard error of skewness; K = kurtosis; SEK = standard error of kurtosis; Maximum score: indicates the maximum score that can be obtained in each of the subscales of the Metacognitive Strategies Scale [42].

**Table 3:** One-factor fixed-effects ANOVA (DGBL use vs. non-use).

Metacognitive Strategies Scale [42]	Occupational Therapy Degree *n* = 98		Nursing Degree *n* = 127		df	F	*p*	η^2^
	Non-Use of DGBL M (SD) *n* = 46	Using DGBL M (SD) *n* = 52	Non-Use of DGBL M (SD) *n* = 61	Using DGBL M (SD) *n* = 66				
Degree								
Self-knowledge	19.58 (3.21)	19.71 (2.33)	20.25 (0.72)	20.42 (2.62)	(1,221)	4.71	0.03 *	0.02
Self-planning	12.50 (2.35)	12.53 (2.26)	12.50 (0.73)	12.57 (2.26)	(1,221)	0.04	0.95	0.00
Self-assessment	19.36 (3.29)	19.28 (2.10)	19.31 (0.52)	19.19 (2.68)	(1,221)	0.05	0.83	0.00
Academic year								
Self-knowledge					(1,221)	0.22	0.64	0.001
Self-planning					(1,221)	0.32	0.86	0.000
Self-assessment					(1,221)	0.10	0.75	0.000

Note: DGBL = digital game-based learning; M = mean; SD = standard deviation; df = degrees of freedom; η^2^ = eta squared effect value; * *p* < 0.05.

**Table 4:** One-factor fixed-effects ANOVA (use of DGBL vs. non-use) in the group of students of occupational therapy.

Metacognitive Strategies Scale [42]	Occupational Therapy Degree *N* = 98		df	F	*p*	η^2^
	Non-Use of DGBL M (SD) *n* = 46	Using DGBL M (SD) *n* = 52				
Access in the LMS	524.28 (231.66)	929.12 (409.57)	(1,96)	35.01	0.00 *	0.27
Average number of visits per day in the LMS	8.32 (3.68)	14.75 (6.50)	(1,96)	35.01	0.00 *	0.27
Learning Outcomes	8.90 (0.48)	8.548 (1.42)	(1,96)	2.60	0.11	0.03
Satisfaction with teaching	4.29 (0.28)	4.24 (0.34)	(1,96)	2.32	0.13	0.02

Note: LMS = learning management system; M = mean; SD = standard deviation; df = degrees of freedom; η^2^ = eta squared effect value; * *p* < 0.05.

**Table 5:** ANOVA of a fixed effects factor (use of DGBL vs. non-use) in the group of nursing students.

Metacognitive Strategies Scale [42]	Nursing Degree *N* = 127		df	F	*p*	η^2^
	Non-Use of DGBL M (SD) *n* = 61	Using DGBL M (SD) *n* = 66				
Access in the LMS	700.49 (237.53)	877.70 (241.83)	(1,125)	17.32	0.00 *	0.12
Average number of visits per day in the LMS	11.12 (3.78)	13.93 (3.84)	(1,125)	17.32	0.00 *	0.12
Learning Outcomes	9.94 (0.30)	9.56 (0.72)	(1,125)	15.19	0.00 *	0.10
Satisfaction with teaching	4.14 (0.33)	4.06 (0.40)	(1,96)	1.28	0.26	0.01

Note: LMS = learning management system; M = mean; SD = standard deviation; df = degrees of freedom; η^2^ = eta squared effect value; * *p* < 0.05.

**Table 6:** Frequencies and percentages in the categorized open-ended responses in bachelor’s degree students of occupational therapy and nursing.

Questions	Categorization Code		Occupational Therapy Degree Answers *N* = 124			Nursing Degree Answers *N* = 125	
		*n*	Frequency	Percentage	*n*	Frequency	Percentage
	All	82	6	0.07	113	6	0.05%
	Crossword		18	21.95%		53	46.90%
	True/false test		58	70.73%		54	47.78%
Question 3	Multiple Choice	21	0	0.00%	8	2	25.00%
	Relationship questions		0	0.00%		3	37.5%
	Speak the Words Set		15	71.42%		1	12.5%
	I would introduce more image play		3	14.29%		2	25.00%
	More activities to work on concepts		3	14.29%		0	0.00%
Question 4	Find the Words	21	21	100%	4	4	100%

**Table 7:** Percentages of feelings (positive, negative and neutral) in the answers to the open questions of the Survey of Satisfaction with the Gamification Activities [48].

Feeling Type	Occupational Therapy Degree Answers *n* = 299		Nursing Degree Answers *n* = 299	
	Frequency	Percentage	Frequency	Percentage
Negative	206	68.90%	177	59.20%
Neutral	12	4.01%	31	10.37%
Positive	81	27.09%	91	30.43%

## Data Availability

The data are available in the repository of the University of Burgos, process in progress.

## Supplementary Materials

The following are available online at https://www.mdpi.com/article/10.3390/ijerph182211757/s1, Table S1: there was an error in the degrees of freedom, they should be separated by commas and not by periods.

## Appendix A

**Table A1:** H5P resources, functionality and type of DGBL.

Resource H5P	Functionality	Type of Serious Games
Accordion	It allows the teacher to create glossaries of key concepts.	conceptual fixation set
Agamotto	Allows the teacher to compare and explore a sequence of images interactively. A short explanatory text can be added for each image.	conceptual fixing and testing set
Branching Scenario	It allows the teacher to create dilemmas, personalized learning scenarios for different learning paces and other types of adaptive learning.	hypothetical-deductive games
Course Presentation	It allows the teacher in their presentations to add multiple choice questions, fill in the blanks, text and other types of interactions using only a web browser.	test sets
Crossword	It allows the teacher to create crossword puzzles	conceptual fixing and testing set
Dialog Cards	It allows the teacher to create learning resources made with images and concept matching in voice.	conceptual fixation set
Drag & Drop	It allows the teacher to create questions and enable answers that are checked by dragging the chosen adoption.	test sets
Drag the Words	It allows the teacher to create a text with blanks so that the user can drag the chosen answer.	test sets
Find images	It allows the teacher to create a set of images with common characteristics.	test sets
Find the words	It allows the teacher to create a grid with different keywords.	conceptual fixing set and testing sets
Interactive video	It allows the teacher to build interactive HTML5-based videos which makes it easy for the user to add multiple choice questions and fill in the blanks, pop-up text and other types of interactions in the videos using just a web browser.	simulation games
Questionnaire	It allows the teacher to create quizzes.	test sets
Memory Game	It allows the teacher to add their own images (and optional text) to a memory game.	
Multiple Choice	It allows the teacher to create multiple choice questions.	test sets
Speak the Words Set	It allows the teacher to create questions that students can answer by voice.	test sets
Timeline	It allows the creative teacher to create interactive timelines.	test sets
True/false questions	It allows the teacher to create true/false questions.	test sets
Virtual tours	It allows the teacher to create a virtual tour that can include questions, texts, interactions in 360 environments from a web browser.	simulation games

**Table A2:** Questionnaire of General Satisfaction with the Training Activity [47].

Items	Rating Scale				
(1) At the beginning of the course, your previous knowledge was at a level of	1	2	3	4	5
(2) At the end of the course, your knowledge is at a level of	1	2	3	4	5
(3) In your opinion, the objectives of the course have been clear.	1	2	3	4	5
(4) In your opinion, the concepts worked on in the course have been clear.	1	2	3	4	5
(5) In your opinion, the practices have helped in the understanding of the theoretical concepts.	1	2	3	4	5
(6) The feedback given by the teacher was quick and accurate.	1	2	3	4	5
(7) In your opinion, group work has been facilitated.	1	2	3	4	5
(8) In your opinion, all the contents specified in the teaching guide have been covered.	1	2	3	4	5
(9) In your opinion, the skills you have developed in this subject can increase your chances of finding a job.	1	2	3	4	5
(10) The expectations you had when you enrolled in this course have been fulfilled.	1	2	3	4	5
(11) In your opinion the procedure and evaluation criteria were clearly explained.	1	2	3	4	5
(12) In your opinion the different assessment tests (internships, project-based learning) facilitated learning	1	2	3	4	5
(13) In your opinion, the use of UBUVirtual as an online teaching platform has been	1	2	3	4	5
(14) In your opinion, the use of questionnaires to evaluate the development of each unit has facilitated the understanding of the unit.	1	2	3	4	5
(15) In your opinion, the difficulty of the subject is at a level of	1	2	3	4	5
(16) Your degree of satisfaction with the development of the practical activities was	1	2	3	4	5
(17) Your degree of satisfaction with the development of the course has been	1	2	3	4	5
(18) In your opinion, with respect to the rest of the subjects taken in the degree, you value this subject.	1	2	3	4	5
(19) Would you recommend this subject to your classmates from other courses?	1	2	3	4	5
	**Open answers**				
(20) Do you think it is convenient to change anything about the subject? Why?					
(21) In your opinion, which units of the current syllabus should be expanded? In theoretical or practical content? Why?					
(22) In your opinion, which units of the current syllabus should be reduced? In theoretical or practical content? Why?					

**Table A3:** Questionnaire of Satisfaction with the Gamification Activities (Sáiz-Manzanares, 2021) [48].

Items	Rating Scale				
(1) The gamification activities have made it easier for me to understand the concepts.	1	2	3	4	5
	Open answers				
(2) Which of the gamification materials have you found most useful for understanding the concepts?					
(3) What elements would you introduce or increase in the gamification materials?					
(4) Which of the gamification elements you would eliminate? Why?					

## References

[1] All A., Castellar E.N.P., Van Looy J. (2021). Digital Game-Based Learning effectiveness assessment: Reflections on study design. Comput. Educ..

[2] Sáiz-Manzanares M., Marticorena-Sánchez R., Muñoz-Rujas N., Rodríguez-Arribas S., Escolar-Llamazares M.-C., Alonso-Santander N., Martínez-Martín M., Mercado-Val E. (2021). Teaching and Learning Styles on Moodle: An Analysis of the Effectiveness of Using STEM and Non-STEM Qualifications from a Gender Perspective. Sustainability.

[3] Lajoie S.P., Pekrun R., Azevedo R., Leighton J.P. (2019). Understanding and measuring emotions in technology-rich learning environments. Learn. Instr..

[4] Nasiri M., Eslami J., Rashidi N., Paim C.P.P., Akbari F., Torabizadeh C., Havaeji F.S., Goldmeier S., Abbasi M. (2021). “Playing with Surgical Instruments (PlaSurIn)” game to train operating room novices how to set up basic surgical instruments: A validation study. Nurse Educ. Today.

[5] Winne P.H., Hadwin A.F., Hacker D.J., Dunlosky J., Graesser A.C. (1998). Studying as self-regulated learning. Metacognition in Educational Theory and Practice.

[6] Pondee P., Panjaburee P., Srisawasdi N. (2021). Preservice science teachers’ emerging pedagogy of mobile game integration: A tale of two cohorts improvement study. Res. Pract. Technol. Enhanc. Learn..

[7] Liu Y.-M., Hou Y.-C. (2021). Effect of multi-disciplinary teaching on learning satisfaction, self-confidence level and learning performance in the nursing students. Nurse Educ. Pract..

[8] Sáiz-Manzanares M.C., Marticorena-Sánchez R., Rodríguez-Díez J.J., Rodríguez-Arribas S., Díez-Pastor J.F., Ji Y.P. (2021). Improve teaching with modalities and collaborative groups in an LMS: An analysis of monitoring using visualisation techniques. J. Comput. High. Educ..

[9] Clark D.B., Tanner-Smith E.E., Killingsworth S.S. (2016). Digital Games, Design, and Learning: A Systematic Review and Meta-Analysis. Rev. Educ. Res..

[10] Pauline-Graf D. (2019). Defining Preliminary Research for Digital Game-Based Learning Evaluation: Best Practices. Int. J. Educ. Methodol..

[11] Whitehead A.L., Sully B.G., Campbell M.J. (2014). Pilot and feasibility studies: Is there a difference from each other and from a randomised controlled trial?. Contemp. Clin. Trials.

[12] Taub M., Azevedo R. (2018). How Does Prior Knowledge Influence Eye Fixations and Sequences of Cognitive and Metacognitive SRL Processes during Learning with an Intelligent Tutoring System?. Int. J. Artif. Intell. Educ..

[13] Taub M., Azevedo R., Rajendran R., Cloude E.B., Biswas G., Price M.J. (2021). How are students’ emotions related to the accuracy of cognitive and metacognitive processes during learning with an intelligent tutoring system?. Learn. Instr..

[14] Geden M., Emerson A., Carpenter D., Rowe J., Azevedo R., Lester J. (2020). Predictive Student Modeling in Game-Based Learning Environments with Word Embedding Representations of Reflection. Int. J. Artif. Intell. Educ..

[15] Green R.D., Schlairet M.C. (2016). Moving toward heutagogical learning: Illuminating undergraduate nursing students’ experiences in a flipped classroom. Nurse Educ. Today.

[16] Sáiz-Manzanares M.C., Zaparain-Yañez M.J., Rodríguez-Arribas S., Bustillo Iglesias A. Design of a Smartart Classroom in Art History: A Learning Experience With Self-Regulated Serious Games. Proceedings of the 13th International Technology, Education and Development Conference.

[17] de Castañeda R.R., Garrison A., Haeberli P., Crump L., Zinsstag J., Ravel A., Flahault A., Bolon I. (2018). First ‘Global Flipped Classroom in One Health’: From MOOCs to research on real world challenges. One Health.

[18] Taub M., Azevedo R., Mudrick N., Clodfelter E., Bouchet F. (2014). Can scaffolds from pedagogical agents influence effective completion of sub-goals during learning with a multi-agent hypermedia-learning environment?. Proc. Int. Conf. Learn. Sci. ICLS.

[19] Thai N.T.T., De Wever B., Valcke M. (2017). The impact of a flipped classroom design on learning performance in higher education: Looking for the best “blend” of lectures and guiding questions with feedback. Comput. Educ..

[20] Schlögl M., Roller-Wirnsberger R.E., Hernes S.S., Perkisas S., Bakken M.S., Miot S., Balci C., Dani M., Pajulammi H., Piaggi P. (2021). Teaching geriatric medicine through gamification: A tool for enhancing postgraduate education in geriatric medicine. Aging Clin. Exp. Res..

[21] Dörner R., Göbel S., Effelsberg W., Wiemeyer J. (2016). Serious Games.

[22] van Gaalen A.E.J., Brouwer J., Schönrock-Adema J., Bouwkamp-Timmer T., Jaarsma A.D.C., Georgiadis J.R. (2020). Gamification of health professions education: A systematic review. Adv. Health Sci. Educ. Theory Pract..

[23] Mackavey C., Cron S. (2019). Innovative strategies: Increased engagement and synthesis in online advanced practice nursing education. Nurse Educ. Today.

[24] Zimmerman B.J., Moylan A., Hacker D.J., Dunlosky J., Graesser A.C. (2009). Self-regulation: Where metacognition and motivation intersect. Handbook of Metacognition in Education.

[25] Kretschmer V., Terharen A., Ahram T.Z. (2019). Serious Games in Virtual Environments: Cognitive Ergonomic Trainings for Workplaces in Intralogistics. Advances in Human Factors in Wearable Technologies and Game Design.

[26] Sáiz-Manzanares M.C., Rodríguez-Arribas S., Pardo-Aguilar C., Queiruga-Dios M.Á. (2020). Effectiveness of Self-Regulation and Serious Games for Learning STEM Knowledge in Primary Education. Psicothema.

[27] Sik-Lanyi C., Szucs V., Shirmohammadi S., Grudeva P., Abersek B., Guzsvinecz T., Van K. (2019). How to Develop Serious Games for Social and Cognitive Competence of Children with Learning Difficulties. Acta Polytech. Hung..

[28] Amali L.N., Kadir N.T., Latief M. (2019). Development of e-learning content with H5P and iSpring features. J. Phys. Conf. Ser..

[29] Chilukuri K.C. (2020). A Novel Framework for Active Learning in Engineering Education Mapped to Course Outcomes. Procedia Comput. Sci..

[30] Churches A. (2009). Bloom’s Taxonomy for the digital age. Eduteka.

[31] Jacobs R.S., Kowert R., Quandt T. (2020). Serious Games: Play for Change. The Video Game Debate 2.

[32] Killam L.A., Luctkar-Flude M. (2021). Virtual Simulations to Replace Clinical Hours in a Family Assessment Course: Development Using H5P, Gamification, and Student Co-Creation. Clin. Simul. Nurs..

[33] Wehling J., Volkenstein S., Dazert S., Wrobel C., van Ackeren K., Johannsen K., Dombrowski T. (2021). Fast-track flipping: Flipped classroom framework development with open-source H5P interactive tools. BMC Med. Educ..

[34] Anguas-Gracia A., Subirón-Valera A.B., Antón-Solanas I., Rodríguez-Roca B., Satústegui-Dordá P.J., Urcola-Pardo F. (2021). An Evaluation Of Undergraduate Student Nurses’ Gameful Experience While Playing A Escape Room Game As Part Of A Community Health Nursing Course. Nurse Educ. Today.

[35] Suppan M., Abbas M., Catho G., Stuby L., Regard S., Achab S., Harbarth S., Suppan L. (2021). Impact of a Serious Game (Escape COVID-19) on the Intention to Change COVID-19 Control Practices Among Employees of Long-term Care Facilities: Web-Based Randomized Controlled Trial. J. Med. Internet Res..

[36] Dacanay A.P., Sibrian J., Wyllie C., Sorrentino E., Dunbar G. (2021). Can You Escape Sepsis? Using a Healthcare Escape Room as an Innovative Approach to Nursing Education. Clin. Nurse Spec..

[37] Garrison E., Colin S., Lemberger O., Lugod M. (2021). Interactive Learning for Nurses Through Gamification. JONA J. Nurs. Adm..

[38] Jiménez-Rodríguez D., Garcia T.B., Arizo-Luque V. (2020). Perception of nursing students about the implementation of GREENS© methodology in nursing studies. Nurse Educ. Today.

[39] Woolwine S., Romp C.R., Jackson B. (2019). Game On: Evaluating the Impact of Gamification in Nursing Orientation on Motivation and Knowledge Retention. J. Nurses Prof. Dev..

[40] Vermeir J.F., White M.J., Johnson D., Crombez G., Van Ryckeghem D.M.L. (2020). The Effects of Gamification on Computerized Cognitive Training: Systematic Review and Meta-Analysis. JMIR Serious Games.

[41] Malicki A., Vergara F.H., Van de Castle B., Goyeneche P., Mann S., Scott M.P., Seiler J., Meneses M.Z., Whalen M. (2020). Gamification in Nursing Education: An Integrative Literature Review. J. Contin. Educ. Nurs..

[42] Román J.M., Gallego S. (2008). ACRA Escalas de Estrategias de Aprendizaje.

[43] Carbonero M.A., Román J.M., Ferrer M. (2013). Program for “strategic learning” with university students: Design and experimental validation. An. Psicol..

[44] Sáiz-Manzanares M.C., Marticorena-Sánchez R., Escolar-Llamazares M.C. (2020). eOrientation Computer Software for Moodle. Detection of the Student at Academic Risk at University.

[45] Sáiz-Manzanares M.C., Marticorena-Sánchez R., García-Osorio C.I. (2020). Monitoring Students at the University: Design and Application of a Moodle Plugin. Appl. Sci..

[46] Ji Y.P., Marticorena-Sánchez R., Pardo-Aguilar C. (2018). UBU Monitor: Monitoring of Students on the Moodle Platform. https://github.com/yjx0003/UBUMonitor.

[47] Sáiz-Manzanares M.C., Marticorena-Sánchez R. (2019). Survey of General Satisfaction with the Training Activity [Encuesta de Satisfacción General Con la Actividad Formativa].

[48] Sáiz-Manzanares M.C. (2021). Survey of Satisfaction with Gamification Activities [Encuesta de Satisfacción con las Actividades de Gamificación].

[49] Campbell D.F., Stanley J.C. (2008). Experimental and Quasi-Experimental Designs for Research [Diseños Experimentales y Cuasiexpe-Rimentales en la Investigación Social].

[50] IBM Corporation (2016). SPSS Statistical Package for the Social Sciences (SPSS), Version 24.

[51] Flick U. (2014). El Diseño de la Investigación Cualitativa [Designing Qualitative Research].

[52] Atlas.ti (2020). Software Package Qualitative Data Analysis, Version 9.

[53] Bandalos D.L., Finney S.J., Marcoulides G.A., Schumacker R.E. (2001). Item parceling issues in structural equation modeling. New Development and Techniques in Structural Equation Modeling.

