# Supplementary material for: Usefulness of Digital Game-Based Learning in Nursing and Occupational Therapy Degrees: A Comparative Study at the University of Burgos
Source: Int J Environ Res Public Health. 2021 Nov 9;18(22):11757. doi: 10.3390/ijerph182211757 (PMC8618151; doi:10.3390/ijerph182211757)

Supplementary Materials

Table S1. Examples of the activities applied in this study with H5P in Moodle.

Gamification activity	Example
Crossword	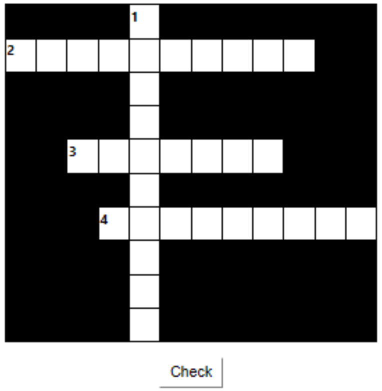
Find the Words	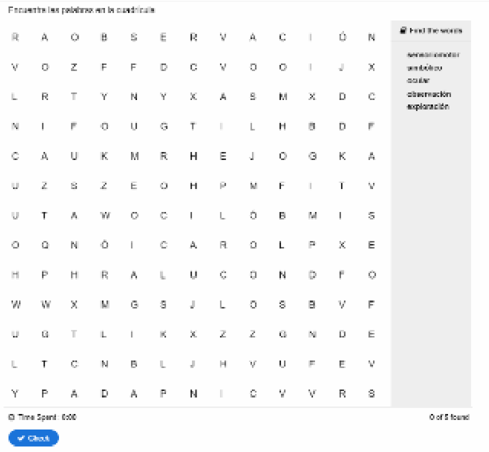

Memory Game

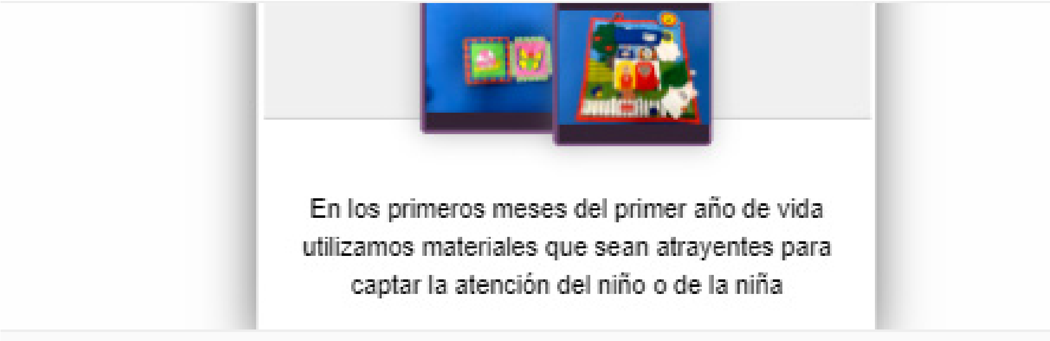

Speak the Words Set

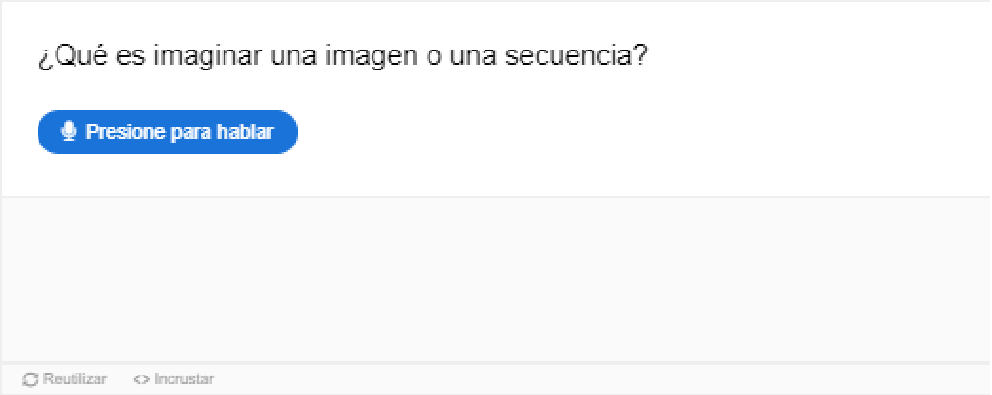

True/False Question

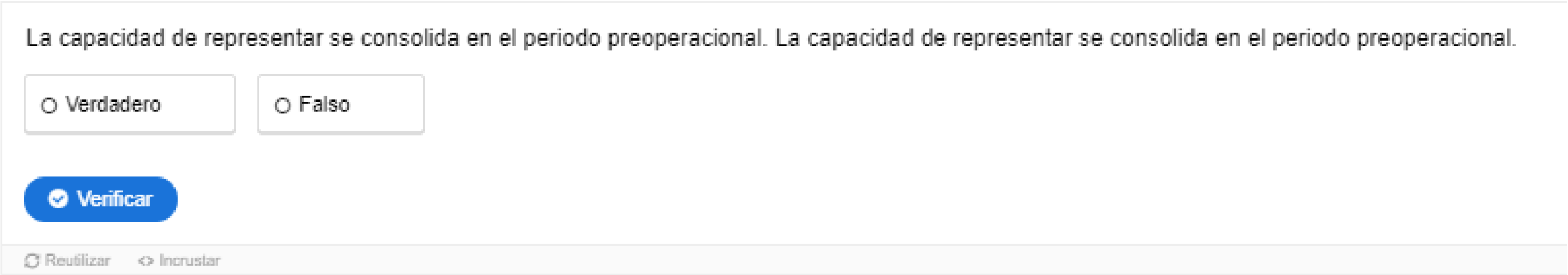

Supplement: Supplementary file 1 [file ijerph-18-11757-s001.zip › ijerph-1413276-supplementary.pdf]
